# Cardiotoxicity after cancer treatment: a process map of the patient treatment journey

**DOI:** 10.1186/s40959-019-0046-5

**Published:** 2019-08-22

**Authors:** Robyn A. Clark, Tania S. Marin, Alexandra L. McCarthy, Julie Bradley, Suchi Grover, Robyn Peters, Christos S. Karapetis, John J. Atherton, Bogda Koczwara

**Affiliations:** 1 College of Nursing and Health Sciences, Flinders University, Adelaide, SA Australia; 2 Acute Care & Cardiovascular Research, College of Nursing and Health Sciences, Flinders University, Adelaide, SA Australia; 3 Faculty of Health and Medical Sciences, The University of Auckland, Auckland, New Zealand; 4 Royal Adelaide Hospital, North Terrace, Adelaide, SA Australia; 5 Flinders Cardiac Clinic, Flinders Private Hospital, Bedford Park, Adelaide, SA Australia; 6 Heart Recovery Service, Cardiology Department, Princess Alexandra Hospital, Ipswich Road, Woolloongabba, Qld Australia; 7 The University of Queensland, St Lucia Campus, St Lucia, Qld Australia; 8 Department of Medical Oncology and Medical Oncology Clinical Research, Flinders Medical Centre, Flinders Drive, Bedford Park, SA Australia; 9 Southern Area Local Health Network, SA Health, Adelaide, Australia; 10 Flinders Centre for Innovation in Cancer, Flinders University, Adelaide, SA Australia; 11 Royal Brisbane and Women’s Hospital, University of Queensland School of Medicine, Butterfield St & Bowen Bridge Rd, Herston, Qld Australia; 12 Flinders Centre for Innovation in Cancer, Flinders Drive, Bedford Park, SA Australia; 13 Flinders University, Adelaide, SA Australia

**Keywords:** Cardiotoxicity, Cardio-oncology, Clinical pathway, Evidence-based practice, Patient centred care

## Abstract

**Background/aim:**

Cardiotoxicity is a potential complication of anticancer therapy. While guidelines have been developed to assist practitioners, an effective, evidence based clinical pathway for the treatment of cardiotoxicity has not yet been developed. The aim of this study was to describe the journey of patients who developed cardiotoxicity through the healthcare system in order to establish baseline data to inform the development and implementation of a patient-centred, evidence-based clinical pathway.

**Methods:**

Mixed-methods design with quantitative and qualitative components using process mapping at 3 large medical centres in 2 states between 2010 and 2015.

**Results:**

Fifty (50) confirmed cases of cardiotoxicity were reviewed (39 medical record reviews, 7 medical record review and interviews and 4 internview only). The mean age at cancer diagnosis of this group was 53.3 years (range 6–89 years); 50% female; 30% breast cancer, 23% non-Hodgkin’s lymphoma; mean chemotherapy cycles 5.2 (median 6; range 1–18); 49 (89%) presented to chemotherapy with pre-existing cardiovascular risk factors; 39 (85%) had at least one modifiable risk factor and 11 (24%) had more than 4; 44 (96%) were diagnosed by echocardiogram and 27 (57%) were referred to a cardiologist (only 7 (15%) before chemotherapy). Post chemotherapy, 22 (48%) patients were referred to a multidisciplinary heart failure clinic; 8 (17%) to cardiac rehabilitation; 1 (2%) to cancer survivorship clinic and 10 (22%) to a palliative care service. There were 16 (34%) deaths during the timeframe of the study; 4 (25%) cardiac-related, 6 (38%) cancer-related, 4 (25%) due to sepsis and 2 (12%) other causes not recorded. The main concerns participants raised during the interviews were cancer professionals not discussing the potential for cardiotoxicity with them prior to treatment, nor risk modification strategies; a need for health education, particularly regarding risks for developing heart failure related to cancer treatment; and a lack of collaboration between oncologists and cardiologists.

**Conclusions:**

Our results demonstrate that the clinical management of cancer patients with cardiotoxicity was variable and fragmented and not patient centered. This audit establishes practice gaps that can be addressed through the design of an evidence-based clinical pathway for cancer patients with, or at risk, of cardiotoxicity.

**Electronic supplementary material:**

The online version of this article (10.1186/s40959-019-0046-5) contains supplementary material, which is available to authorized users.

## Background

Cardiotoxicity resulting in cardiac complications can be a devastating consequence of cancer therapy. It is possible that a patient may survive cancer only to develop heart failure (cardiomyopathy), which has a higher mortality rate than cancer [1, 2]. Treatment-induced cardiotoxicity (particularly cardiomyopathy), defined as the direct effects of cancer treatment on heart function and structure [3, 4] occurs in up to one quarter of cancer patients (depending on their underlying cardiovascular risk factors and the chemotherapeutic agent administered) [1], although current figures are probably underestimates [5, 6]. For example, survivors of childhood cancers (who are increasingly reaching adulthood) have an eightfold risk of cardiac-related mortality [7, 8]. The incidence rates of cardiotoxicity from commonly-prescribed chemotherapy agents most frequently associated with this adverse outcome include anthracyclines (1–26%) [1, 5, 7], high-dose cyclophosphamides (7–28%) [1, 7, 9, 10], trastuzamab (2–28%) and increasingly, tyrosine kinase inhibitors. (0.05–11%) [1, 7] There are also many cancer drugs emerging from clinical trials with similar cardiotoxic effects [2]. This adverse effect of cancer therapy, which can occur up to 20 years after treatment [5, 11, 12] is likely to become even more prevalent as the cancer population ages as a result of extended cancer-free survival with an increasing number of novel chemotherapeutic approaches associated with cardiotoxicity. Less well recognised, but equally important in blood, breast, and lung cancers, is the role of concomitant chest irradiation in inducing cardiotoxicity [13, 14]. Fortunately, it is possible that with better initial monitoring and intervention during cancer treatment, and continued surveillance after treatment, cardiotoxicity can be prevented or ameliorated [1, 3, 11, 13, 15–17]. Unfortunately, the clinical trials upon which the modern management of heart failure were developed uniformly excluded patients with a history of cancer; thus evidence-based guidelines specific to the assessment, monitoring and management of patients with cardiotoxicity have been slow to appear [3, 18–21]. At present, only a limited number of the possible risk factors associated with this toxicity are well-understood [1, 3, 7, 11, 13, 15–17, 22–25].

A good understanding of cardiotoxicity risk factors and cardiotoxicity patients’ service needs at baseline, through to early detection and optimal management strategies in the acute and survivor stages could enhance the safety of anticancer treatments.

The effectiveness of clinical pathways to improve clinical outcomes is well established [26]. The aim of a clinical pathway is to enhance the quality of care across the continuum by improving risk-adjusted patient outcomes, promoting patient safety, increasing patient satisfaction, and optimizing the use of resources. A clinical pathway is a complex intervention for the mutual decision-making and organisation of care processes for a well-defined group of patients during a well-defined period. Defining characteristics of care pathways include: an explicit statement of the goals and key elements of care based on evidence, best practice, and patients’ expectations and their characteristics; the facilitation of the communication among the team members and with patients and families; the coordination of the care process by coordinating the roles and sequencing the activities of the multidisciplinary care team, the patients and their relatives; the documentation, monitoring, and evaluation of variances and outcomes, and the identification of the appropriate resources. An enhanced clinical pathway may also help to ensure that extended cancer-free survival in patients with cardiotoxicity translates to improved survival and overall health [26].

Patient-centred care is health care that is respectful of, and responsive to, the preferences, needs and values of patients and consumers. The widely accepted dimensions of patient-centred care are respect, emotional support, physical comfort, information and communication, continuity and transition, care coordination, involvement of family and carers, and access to care. Surveys measuring patients’ experience of health care are typically based on these domains. Research demonstrates that patient-centred care improves patient care experience and creates public value for services. When healthcare administrators, providers, patients and families work in partnership, the quality and safety of health care rises, costs decrease, and provider satisfaction increases and patient care experience improves. Patient-centred care can also positively affect business metrics such as finances, quality, safety and satisfaction [27].

It is also clear that good linkages between oncology and cardiology, are essential to ensure best practice in this area [3, 28, 29]. While guidelines have been developed to assist practitioners [3, 28], an effective, evidence-based clinical pathway for the treatment of cardiotoxicity has not yet been developed and access to specialist cardio-oncology care in most countries is very limited.

The aim of this study was to describe the journey of patients who developed cardiotoxicity through the healthcare system in order to establish baseline data to inform the development and implementation of a patient-centred, evidence-based clinical pathway.

The specific objectives were to:

1. Describe the diagnosis, referral and ongoing care a group of cancer patients diagnosed with cardiotoxicity through a medical record review
2. Augment the medical record reviews with interview data that provided patient experiences of the clinical management of their cardiotoxicity
3. Describe practice gaps and make recommendations for clinical practice that can be addressed through a clinical pathway for effective cardio-oncology care.

## Methods

### Study design

This study used a mixed-methods design with quantitative and qualitative components using process mapping [30]. Healthcare Process Mapping is a new and important form of clinical audit that examines how we manage the patient journey, using the patient’s perspective to identify problems and suggest improvements. Process Mapping allows us to “see” and understand the patient’s experience by separating the management of a specific condition or treatment into a series of consecutive events or steps (e.g. activities, interventions, staff interactions). Process Mapping has shown clinical benefit across a variety of specialties, multidisciplinary teams, and healthcare systems. Our Process Mapping included medical recorded review augmented with in-depth, semi-structured, one-to-one interviews [28]. There were 3 important timeframes described in this study 1) Cancer diagnoses (1979–2015); Cardiotoxicity diagnoses (1994–2015); and the timeframe in which the study was conducted 2010–2015.

### Setting

The study was conducted at three large metropolitan medical centres in two separate states of Australia. Each centre was greater than 600 beds with tertiary level cardiac and cancer services.

### Participants and inclusion criteria

Participants were identified by their treating cardiologists at each of the three sites. Echocardiography and Multi Gated Acquisition Scans (MUGA) imaging databases at each of the three sites were searched for the following inclusion criteria: documented diagnosis of cardiotoxicity; chemotherapy or chest radiotherapy treatment for any cancer. Patients who received targeted therapy and immunotherapy were not excluded. Participants were considered to have cardiotoxicity if there had been a LVEF reduction of ≥15% from baseline despite normal function, or an LVEF decline to < 50%, or an LVEF decline considered clinically significant by the cardiologist (as per the 2012 European Society for Medical Oncology (ESMO) Clinical Practice Guidelines, available at the time of the medical record review) [28].

Patients interviewed all had undergone breast or blood cancer (leukemia, non-hodgkin lymphoma, hodgkin lymphoma, multiple myeloma) treatment including surgery, radiotherapy and/or chemotherapy, and were diagnosed with heart failure (HF) which had been confirmed by the referring cardiac specialist as cardiotoxicity related to cancer treatment.

### Medical record review variables

Variables collected from the medical records included: demographics, anthropometrics, family history, cancer diagnoses and history, cardiac risk factors (pre-and post-chemotherapy), cardiotoxicity diagnosis and treatment (pharmacotherapy, internal cardiac defibrillator, biventricular pacing etc) and health systems access, retrospectively from the patient’s cancer diagnosis through to the day of the audit. The sociodemographic data collected informed the data analysis process and provided an epidemiological population health view of the participants.

### Data collection

A data extraction tool (DET) (Additional file 1) was developed based on cardiotoxicity guidelines available at the time of the audit [26]. Two research assistants undertook the manual data extraction and a third senior researcher undertook a random quality check on 10 % of the cases. All data were de-identified. The first five medical records reviewed were undertaken as a pilot to test the DET. The DET was reformatted to follow a more streamlined approach that matched the medical record. The final DET was then used for the remainder of the data extraction at all three sites.

Qualitative data were collected via in-depth, semi-structured, one-to-one interviews. An interview schedule (Additional file 2) consisting of 49 items was constructed for the purposes of (a) assessing participants’ cancer and cardiac history as well as modifiable and non-modifiable cardiac risk factors and (b) acquiring a patient-centred perspective on the knowledge, experiences and health service needs of individuals with cardiotoxicity**.** Specifically, the questionnaire sought to determine patients’: knowledge and experience of their heart condition/treatment; knowledge and experience of their cancer condition/treatment; awareness of the cardiac risks associated with their anti-cancer therapy; experience of and access to cardiac care services; state of overall health since developing cardiotoxicity; satisfaction with oncological and cardiac services; and perceived health care needs. The interviews ran for approximately 1 h and were undertaken by two experienced qualitative nurse researchers in the patient’s home or at their usual treatment location.

### Bias

Bias was minimised by the third person cross check and assessment of selected cases to ensure quality. Other team members reviewed the coding of the data and outcomes between the two sources.

### Statistical methods and analysis

To achieve Objective 1, quantitative data were entered into Microsoft® Excel 2011 and IBM® SPSS® Version22 for Windows for analysis. A profile of participants was generated using frequency and summary descriptive statistics. Categorical data is presented here as frequencies, and continuous variables as means and standard deviations for normally distributed data. The sociodemographic data collected informed the data analysis process and provided an epidemiological population health view of the participants. As this was a small sample size and data was collected by specialist research nurses there was limited to no missing data amongst the variables. Most missing data was related to poor documentation in the medical records and therefore was coded as not recorded.

To achieve Objective 2, qualitative data from all interviews were transcribed for analysis and read and re-read to achieve overall familiarity with the material and gain a broad understanding of the experiences of patients with cardiotoxicity in the Australian health care system. This was followed by a more deductive analysis of the aspects of patient experience that were of primary interest to the research team (i.e. knowledge and experience of their cancer and heart conditions/treatment, risk awareness, experience of cardiac care services, state of overall health, satisfaction with oncological and cardiac services and perceived health care needs). Key aspects of cardiotoxicity-patient knowledge and experience were identified. These included: Risk awareness associated with heart care; Access to health services and other support; Physical activity and nutrition; Overall health after HF; Knowledge of diagnostic procedures and Behavioural risk factors. Representative quotations were then extracted and collated (See Table 1).

**Table 1:** Patient interview quotes

Risk awareness associated with heart care	
“… Yeah there was no mention that it could lead to, in let’s say 20% of people, lead to heart disease or anything like that, no. Would have been nice to know.”	
“… they have a couple of cardiac tests, being BMP and TN, and the nursing staff don’t actually even know what they are. So that’s a little bit of an indication I think that they’re not aware of the heart side of things.”	
Access to health services and other support	
“… Even the dietician they ring me up at home, and they are really happy.”	
“… The rehab exercise program has an information session every week and they go through different areas, and that’s really informative and there is usually people in the group that have been through, so are hearing other people’s experiences.”	
“… Oncologist is brilliant, and the neurologist is good. I have been very fortunate, happy with hospital services.”	
Physical activity and nutrition	
“… because I have been through too many episodes where I crash and burn trying to do too much, and then suffer for it for 2 to 3 days. Now I try and pace myself.”	
“… I didn’t have breakfast and I really stuck to it; and very rarely I’d have lunch”.	
“… I am not a big fruit eat eater; I always have two meals a day instead of three. I think my eating habits are healthy because I have cut down on red meat and carbohydrates.”	
Overall health after HF	
“… even up a ramp, I’ll lose my breath … and I start to get tired and physically just go down, completely down, in just a few minutes”.	
“… I feel like a 90-year-old ... Yeah, I’ve got no energy. If I do anything, I get out of breath really quick.”	
Knowledge of diagnostic procedures	
“That’s the sticky one, that’s not the echo. ECG is when they put the sticky things on your chest and take a tracing of your heart.”	
“Oh yeah, they stuck some sticky things on me, a little while ago.”	
Behavioural risk factors	
“I stay away from beer ‘cause it’s got hops in it which has got oestrogen in it and that feeds the … cancer. Um, I might drink one beer once a fortnight, maybe two and then I might have on the average two cans of rum and coke …”	
“I know I shouldn’t be ... well it’s one packet every 5 or 6 days … the patches weren’t working ... I had to go off that because of the other tablets I was on.”	

To achieve Objective 3, team members reviewed the data and triangulated the outcomes between two sources of data.

### Ethics

Ethics approvals covering the three sites were provided by the Clinical Human Research Ethics Committee (HREC) (#181.15); Metro South Health Service District HREC (#225.15), and Queensland University of Technology HREC (#445.15).

## Results

### Participants

**Fig. 1:**
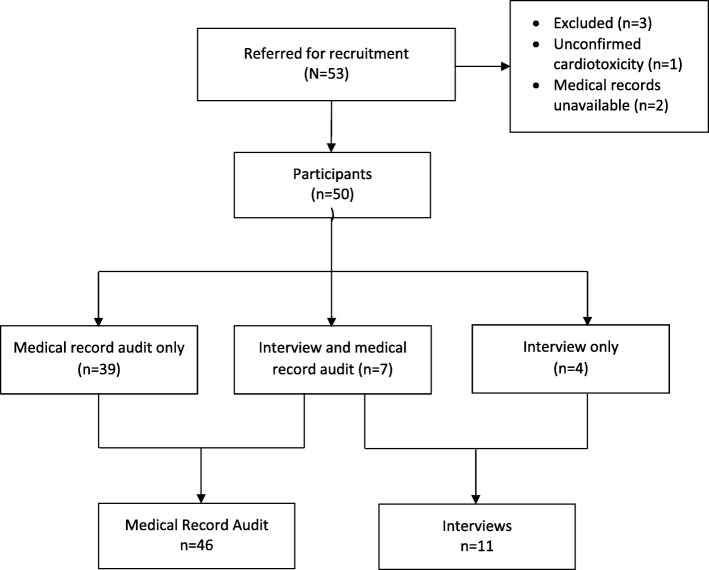
Study flow chart

Fifty-three participants were identified for the study with three not meeting inclusion criteria (two patients’ records were not available, and one patient did not meet cardiotoxicity criteria). Of the 50 participants who were eligible to take part in the study, 39 were included in the medical record review, 7 were included in both medical record review and interviews (total 46 medical record reviews) and 4 undertook interview only (total 11 Interviews). Figure 1 provides an overview the screening and inclusion process.

### Demographics and characteristics

Table 2 presents the key demographic and clinical characteristics of the cases reviewed. The mean age of the participants was 58.5 years, 23 (50%) were female. 75% identified as Australian; 90% had one or more pre-existing cardiovascular risk factor. Almost 40% were overweight or obese; 24% had a family history of CVD, 41% were current or ex-smokers, 24% were regular consumers of alcohol, 48% had hypertension and 26% diabetes. A quarter of patients presenting for cancer treatment (24%) had more than four cardiovascular risk factors.

**Table 2:** Participant demographics, baseline clinical characteristics and pre-existing cardiovascular risk factors (Medical Record Review cohort only)

	*N* = 46 n (%)
Demographics	
Female	23 (50)
Married/de facto	28 (61)
Country of birth (Australia)	32 (70)
Private health insurance	15 (33)
Mean age of cardiotoxicity diagnosis (years)	58.5
Baseline clinical characteristics (pre-existing risk factors)	
≥ 1 risk factor	41 (90)
≥ 4 risk factors	11 (24)
Diabetes (type 1 or 2)	12 (26)
Hypertension	22 (48)
Hypercholesterolemia	14 (30)
Stroke	1 (2)
Angina	5 (11)
Arrhythmia (atrial fibrillation)	6 (13)
Valvular disease	1 (2)
Current−/ex-smoker	19 (41)
Social drinker	11 (24)
Overweight/obese	18 (39)
Family history of cardiovascular disease	11 (24)
Participants with four or more risk factors	11 (24)

### Cancer and treatment

Nineteen (57%) cancers diagnosed were haematological other cancers included breast (30%) and Prostate (2%). Details of chemotherapy agents and mean doses are also presented in Table 3. The most common agents included Cyclophosphamide 48%; Doxorubicin 39%; Monoclonal Antibodies 39%; Vincristine 26% and Methotrexate 11%. Sixty percent of the cases had received radiotherapy, 32% directly to the chest. Radiotherapy doses were not routinely recorded in medical records.

**Table 3:** Cancer diagnosis/type, chemotherapy agent class and radiation therapy (Medical Record Review cohort only)

*N* = 46	Mean (range)	n (%)
Cancer diagnosis		
Age at first cancer diagnosis (years)	53.6 (6.3–89.3)	46 (100)
Bile duct		1 (2)
Breast		14 (30)
Colon		1 (2)
Hodgkin’s lymphoma		1 (2)
Leukaemia		3 (7)
Lung		1 (2)
Lymphoma		7 (15)
Non-Hodgkin’s		11 (24)
Oesophagus		1 (2)
Osteosarcoma		4 (9)
Prostate		1 (2)
Missing		1 (2)
Agent Class		
Chemotherapy cycles^b,c^	5.2 (1–18)	
Alkylating	Mean dose (mg)	
Carboplatin	520.0	2 (4)
Chlorambucil^a^		1 (2)
Cisplatin	113.3	5 (11)
Cyclophosphamide	1148.1	21 (48)
Anthracyclines		
Doxorubicin	462.1	18 (39)
Epirubicin	164.0	2 (4)
Anthracycline combination		
ABVD^a^		1 (2)
Antibiotic cytotoxics		
Bleomycin	20.0	1 (2)
Antimetabolites		
ATRA Azacitidine therapy	100.0	1 (2)
Adjuvant CTx with carboplatin/gemcitabine^a^		1 (2)
Fluorouracil	980.0	3 (7)
Methotrexate	63.0	5 (11)
Fludarabine phosphate^a^		1 (2)
Mitotic Inhibitors		
Amsacrine	140.0	1 (2)
Docetaxel	145.7	4 (9)
Etoposide	200.0	1 (2)
Paclitaxel	429.0	3 (7)
Vincristine^a^		12 (26)
Monoclonal Antibodies		
Rituximab	655.0	11 (24)
Trastuzumab	378.1	7 (15)
Radiation therapy		28 (61)

^a^ Dose not recorded

^b^ Each patient’s regimen comprised individualised composites and doses of the above cytotoxic agents

^c^ Other drugs taken include: amiloride, amiodarone, amlodipine, atenolol, atorvastatin, bisoprolol, candesartan, carvedilol, cephalexin, cholecalciferol, clonidine, clopidogrel, digoxin, diltiazem, enalapril, enoxaparin, eprosartan, exemestane, frusemide, ibandronate, irbesartan, itraconazole, ivabradine, letrozole, lisinopril, metoprolol, telmisartan/hydrochlorothiazide, nebivolol, olmesartan, oxycodone, pantoprazole, perindopril, prazosin, prednisolone, ramipril, rivaroxaban, simvastatin, sotalol, spironolactone, tamoxifen, tazocin, goserelin zoledronic acid and vancomycin

### Mapping the cardiotoxicity patient journey: diagnosis, referral, treatment and monitoring

**Fig. 2:**
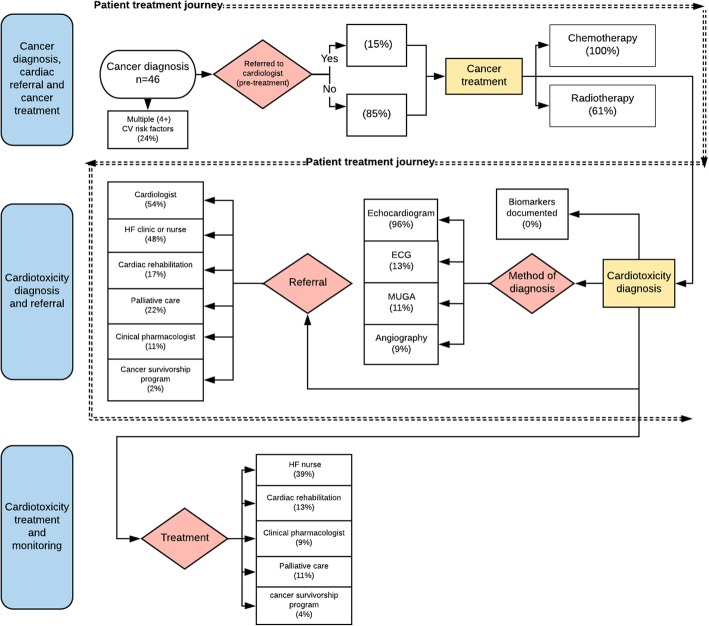
Process Map of the journey of the patients with cardiotoxicity through the healthcare system

The Process Map (Fig. 2) describes the treatment journey of patients diagnosed with cardiomyopathy from cardiotoxicity after cancer treatment. Only 15 % of cases were referred to a cardiologist prior to cancer treatment (24% had more than 4 cardiovascular risk factors), with 43% referred after cardiotoxicity diagnosis or onset of clinical signs and symptoms. The primary form of diagnosis of cardiomyopathy from cardiotoxicity was echocardiogram (ECHO) (96%), with a smaller proportion of patients receiving a multigated acquisition (MUGA) (11%) and angiography (9%) (which was appropriate for those cases). No cases had documentation of cardiac biomarkers such as troponin or brain natriuretic peptide (BNP).

After diagnosis of cardiotoxicity, 48% of cases were referred to a multidisciplinary HFclinic, 17% to cardiac rehabilitation, 11% to a clinical pharmacologist, 2% to a cancer survivorship program and 22% to palliative care. In terms of management, 39% of those diagnosed with cardiotoxicity saw a HF nurse, 13% attended cardiac rehabilitation, 9% saw a clinical pharmacologist, 11% received palliative care and 4% attended a cancer survivorship program. Heart rate was monitored in 44% of cases and blood glucose and HbA1c levels were monitored in 22 and 2% of cases respectively. Table 4 presents information on diagnosis, referral, treatment and monitoring in tabular form. There were 16 deaths (35%) during the timeframe of the study. Four of these (25%) were cardiac-related, 6 (38%) were cancer-related, 4 (25%) were due to sepsis and 2 (13%) were not recorded.

**Table 4:** Cardiotoxicity diagnosis, management, monitoring and referral to cardiac or palliative care (Medical Record Review cohort only)

Cardiotoxicity diagnosis and management	*N* = 46
Referred to cardiologist (any)	27 (59)
Referred to cardiologist prior to first chemotherapy	7 (15)
Method of cardiotoxicity diagnosis^a^	
ECG	6 (13)
MUGA	5 (11)
Echocardiography (LVEF < 53%)	44 (96)
Angiography	4 (9)
Biomarkers	Not documented
Monitoring during and after anticancer therapy	
HbA1c	1 (2)
Glucose	10 (22)
Systolic Blood Pressure (mmHg^b^)	24 (52)
Diastolic Blood Pressure (mmHg)	22 (48)
HR (beats per minute)	20 (44)
Heart failure multidisciplinary care/cardiac rehabilitation	
Referred to HF clinic or nurse	22 (48)
Saw a HF nurse	18 (39)
Referred to cardiac rehabilitation	8 (17)
Attended cardiac rehabilitation	6 (13)
Referred to clinical pharmacologist	5 (11)
Saw a clinical pharmacologist	4 (9)
Cancer survivorship or palliative care	
Referred to palliative care	10 (22)
Received palliative care	5 (11)
Cancer survivorship program referred	1 (2)
Cancer survivorship program seen	2 (4)

^a^ Some patients had more than one diagnostic procedure

^b^ Millimeter of mercury

### Interviews

The main concerns participants raised during the interviews were (a) cancer professionals not discussing the potential for cardiotoxicity with them prior to treatment, nor risk modification strategies (b) a need for health education, particularly regarding risks for developing HF related to cancer treatment; and (c) a lack of collaboration between oncologists and cardiologists (See Table 1, *Risk awareness associated with heart care*). When asked about the risks associated with their heart, the participants appeared to be poorly informed. Pronouncing or remembering the names of heart diagnostic tests emerged as a potential barrier to communicating effectively with their healthcare providers, family, and peers (See Table 1, *Knowledge of diagnostic procedures*).

Several participants were not sure whether they had ever had an ECG or Echo. More than half (60%) of the participants emphasised that they had adopted healthy eating habits after their cancer diagnosis; however, appeared to lack an understanding of what constituted a balanced diet for heart health. Others reported exercise intolerance and three participants linked this to their shortness of breath and fatigue (See Table 1, *Physical activity and nutrition*). Interviewees noted a change in their overall health after the diagnosis of their HF and compared signs of health deterioration with their overall health before HF (See Table 1, *Overall health after HF*). Formal services such as support groups, were reported generally as limited, or for some participants, non-existent. Where they were available, they were valued (See Table 1, *Access to health services and other support*). Several behavioural risk factors were also identified (See Table 1, *Behavioural risk factors*). Of the participants who were interviewed, none could clearly articulate their heart healthcare needs and more than half lacked knowledge of their family history of cardiovascular disease (CVD), diabetes and high cholesterol.

### Impact of guidelines on practice (ESMO 2012)

Table 5 presents a sub-analysis of the 45 cases with most accurately documented treatment who were diagnosed pre-and-post publication of the first practice guidelines for cardiotoxicity [28]. This analysis shows increases over time, in referral to cardiologist pre-cancer care (37%), increased referral to cardiology at any time (4%), increases in diagnosis by ECHO (15%) and decreases in death in the study cohort (11%).

**Table 5:** Cardiotoxicity treatment: pre-and-post 2012 ESMO Guidelines [26]

Date of cardiotoxicity diagnosis	1994–2011 (*n* = 14)^a^	2012–2015 (*n* = 31)^a^	Change	*P* value
Referred to cardiologist (pre-chemotherapy)	0 (0%)	7 (37%)	↑ 37%	0.060
Referred to cardiologist (any)	8 (57%)	19 (61%)	↑ 4%	0.793
Baseline ECHO undertaken	8 (57%)	23 (72%)	↑ 15%	0.253
Died during study period	6 (43%)	10 (32%)	↓ 11%	0.492

^a^ Values and Percentages based on 45 of the 46 cases reviewed due data unavailability

## Discussion

This study described the clinical management of 50 patients with a confirmed diagnosis of cardiomyopathy from cardiotoxicity between 1994 and 2015. During this period, 39 (85%) of the patients studied entered their cancer therapy with at least one CVD risk factor including hypertension, current smoking, high body mass index (BMI), hypercholesterinemia, diabetes and age over 65 years, with 24% having four or more modifiable CVD risk factors. This profile is consistent with current levels of CVD risk factors in the Australian population [16]. New guidelines [3, 28] have clearly indicated that pre-existing risk factors contribute to a high risk of cardiotoxicity and that high risk patients should be referred to a cardiologist or, if necessary, a cardio-oncology specialist team for evaluation at the beginning of cancer treatment [3].

In the medical record review, it was found that half (54%) of the participants were referred to cardiology services when cardiotoxicity was diagnosed. However, while almost all participants were found to have had an ECHO following cardiotoxicity diagnosis, the majority were prescribed by the oncologist and not a cardiologist. New guidelines recognise the need for a ‘dynamic partnership’ between oncologists and cardiologists; along with the development of risk management plans which are a product of explicit and organised collaboration between specialists from the two fields [28].

Only seven participants (15%) were referred to a cardiologist prior to chemotherapy (see Fig. 2, Tables 4 and 5). However, all of those who were referred to a cardiologist pre-chemotherapy were referred after the publication (2012) of clinical practice guidelines [28] for the treatment of cardiotoxicity. This finding may suggest that the guidelines are having a positive impact on patient care - lending further weight to the argument that a robust, evidence-based clinical pathway is likely to further improve patient outcomes.

In addition to the medical record reviews (MRR) of patient clinical care, our team believed it was essential that we should augment or triangulate the MRR data with Patient Reported Experiences (PREMs) [31] so that all outcomes from the study would remain patient-centred. Interview data supported the MRR data with some participants raising concerns about lack of collaboration between oncologists and cardiologists. Challenges in communication among specialists, especially those in different institutions, along with excessive compartmentalisation can easily result in fragmented care. Indeed, multispecialty groups incorporating cardiovascular and cancer care under one health care umbrella are rare [32]. Ewer and Ewer [33] argue that to maximize both overall health and survival, cardiologists and oncologists should collaborate with the aim of balancing the risks of cardiotoxicity with the benefits of oncologic therapy. Other concerns from participants included a lack of knowledge and/or information about risk factors for HF and preventive measures. They wished to be made aware of the possibility of developing HF before commencing cancer treatments even if the chances where minimal.

Cardiotoxicity has previously been classified as early-onset (during therapy or within the first year after treatment), late-onset (at least 1 year after completion of treatment), and late-occurring (10–20 years after first treatment) [28]. More recent recommendations [3] do not attempt to classify. Our study shows that in most cases, participants were diagnosed with their cardiotoxicity within a year of having treatment, while most of the remaining were late-onset (1–7 years).

The methods of diagnosis were appropriate for the time frame of this study and it was encouraging to see that 95% of patients had an echocardiography [34]. The method of diagnosis in this study was based on an LVEF reduction of ≥15% from baseline despite normal function, or an LVEF decline to < 50%, or an LVEF decline considered clinically significant by the cardiologist (as per the 2012 European Society for Medical Oncology (ESMO) Clinical Practice Guidelines, available at the time of the medical record review). The European Society of Cardiology (ESC) Guidelines from 2016 [3] have now updated the diagnostic criteria for echocardiogram and stated that echocardiography is the method of choice for the detection of myocardial dysfunction before, during and after cancer therapy. Unless three-dimensional (3D) ECHO is used, which is the best echocardiographic method for measuring LVEF when endocardial definition is clear, the two-dimensional (2D) biplane Simpson method is recommended for estimation of LV volumes and ejection fraction in these patients. Cancer therapeutics–related cardiac dysfunction (CTRCD) is defined as a decrease in the LVEF of > 10 percentage points, to a value below the lower limit of normal. This decrease should be confirmed by repeated cardiac imaging done 2–3 weeks after the baseline diagnostic study showing the initial decrease in LVEF. The LVEF decrease may be further categorized as symptomatic or asymptomatic or reversible. Although, the exact interval is not established, echocardiographic examination should be repeated during follow-up to confirm recovery, or to detect irreversible LV dysfunction. Echocardiography can also detect other complications of cancer therapy, including valvular and pericardial diseases and findings suggestive of pulmonary hypertension. The main limitation of 2D echocardiography is its relatively moderate reproducibility, which can be improved by the use of 3D echocardiography. The latter is associated with the best day-to-day reproducibility, but remains dependent on image quality, availability and operator experience. For serial evaluation of patients with cancer, LVEF measurements should ideally be performed by the same observer with the same equipment to reduce variability.

There was no reported testing for biomarkers for cardiac function despite evidence of this being a more specific tool for early, real-time identification, assessment and monitoring of cardiotoxicity [35] and its inclusion in the (2012) Guidelines [28]. The established biomarkers included troponins (Tn I and T) and naturetic peptides (BNP and NT-pro BNP), however there are a multitude of biomarkers of carditoxicity that have been investigated in a preliminary manner. Athough biomarkers have shown utility, their routine use for surveillance is yet to be established [3, 4, 28].

Referral to cardiac care after cardiotoxicity diagnosis occurred in less than 60% of patients and referral to heart health care, cardiac rehabilitation, and HF management programs or cancer survivorship programs after cancer treatment was even lower. The time from cancer diagnosis to cardiotoxicity in this cohort was between 0 and 18 years with mean time to death of 5 years for 16 patients. Mortality from cardiotoxicity has been demonstrated to be higher than both cancer and CVD alone [36].

Compromised independence and overall health, associated with the ongoing exercise intolerance and impaired ability to adequately engage in activities of normal daily life, was reported in interviews. According to Hughes and colleagues [37], supervised exercise has been found to be beneficial in HF patients and in cancer survivors without HF. While the same benefits have not been tested in cancer survivors with treatment-induced HF because cancer survivors with HF are usually ineligible to participate in studies due to exclusion criteria; it is reasonable to extrapolate that medically supervised exercise would be beneficial. Interview participants discussed their lifestyle and factors influencing their behaviour, with the discussion purposely focused by the researchers upon diet, exercise, smoking and alcohol intake. Fatigue typical for some time after cancer treatment, coupled with incipient or actual HF, appeared to impede patients’ will or ability to undertake the recommended healthy lifestyle that could prevent or manage the risk of HF.

In terms of reinforcing factors, most of the participants gained some satisfaction from using the public healthcare system. These participants stressed the positive and encouraging aspect of the engaged and caring healthcare workers. Another source of reinforcement originated in support groups, family and friends. All participants, except for one woman who was a widow, reported social reinforcement that was mostly centred on their spouses.

### Recommendations for clinical practice and research

The challenges for managing cancer treatment induced cardiotoxicity call for further concerted research. We would recommend a strategy that could better stratify risk and identify patients in which primary prevention or secondary prevention would be the most beneficial as a priority including more studies on the predisposing factors for the development of CVD related to cancer treatment. What is also needed are studies examining the reversibility of subclinical LV dysfunction preventing its transition to overt HF. The most reliable cardiac monitoring approach needs to be defined.

Comparing clinically relevant outcomes with genetic, epigenetic, biomarker and imaging characteristics assessed at baseline and during active cancer treatment could provide data that would allow the construction of true evidence-based strategies and open a new era in cardio-oncology. Pharmaceutical companies must prioritise the elimination of cardiovascular side effects from all new chemotherapies.

### Limitations

We acknowledge that the first available clinical guidelines for cardiotoxicity did not emerge until 2012 and many of the cases examined occurred before this date. As is well known, the quality of clinical documentation in both paper-based and electronic medical records continues to be of variable quality. Missing data (documentation) should be considered a limitation in this study and this has implications for the generalisability of these data but also may underestimate treatment if data were not centralised in the medical records, for example details of radiation therapy including dose and frequency. While our study examined clinical management in three different settings these sites are not necessarily representative of clinical management in all hospitals. All cases for both medical review and interview were purposefully selected which may have introduced some bias in the sample.

## Conclusions

This paper is the first to describe the clinical management of a group of patients with a confirmed cardiotoxicity diagnosis using a process map. The data collected provides an important baseline against which to measure the effectiveness of future cardiotoxicity treatment strategies. The results demonstrate that the care of patients with cardiotoxicity was both variable and fragmented, and not patient-centred over the period examined and that a clear, evidence based clinical pathway is needed in order to improve treatment for those with, or at risk of, cardiotoxicity.

## Additional files

Additional file 1: Data extraction tool (DET) – MRR. (DOCX 206 kb)

Additional file 2: Interview schedule. (DOCX 16 kb)

## Data Availability

The datasets used and/or analysed during the current study are available from the corresponding author on reasonable request.

## Abbreviations

BMI: Body mass index
CVD: Cardiovascular disease
DET: Data extraction tool
ECG: Electrocardiogram
ECHO: Echocardiogram
ESC: European Society of Cardiology
ESMO: European Society for Medical Oncology
FMC: Flinders Medical Centre
HF: Heart failure
HREC: Human Research Ethics Committee
LVEF: Left ventricular ejection fraction
MRR: Medical record review
MUGA: Multi Gated Acquisition Scan
PAH: Princess Alexandra Hospital
RAH: Royal Adelaide Hospital
