# Supplementary figures and images for: Cardiotoxicity after cancer treatment: a process map of the patient treatment journey
Source: Cardiooncology. 2019 Aug 22;5:14. doi: 10.1186/s40959-019-0046-5 (PMC7048085; doi:10.1186/s40959-019-0046-5)

**Additional file 1: Data extraction tool (DET) - MRR**

**
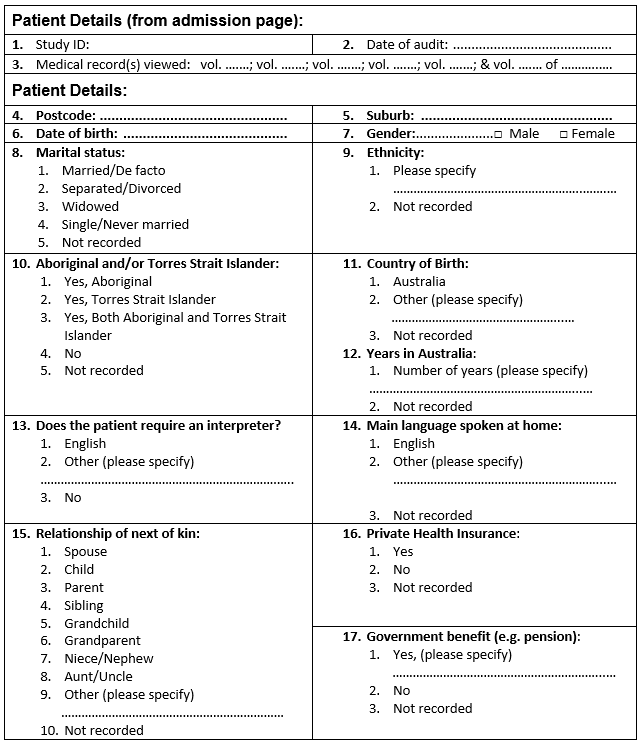
**

**
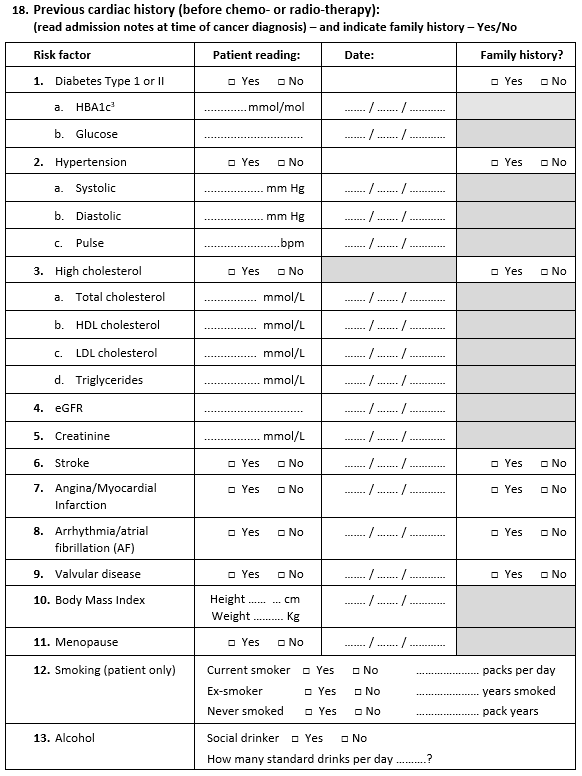
**

**
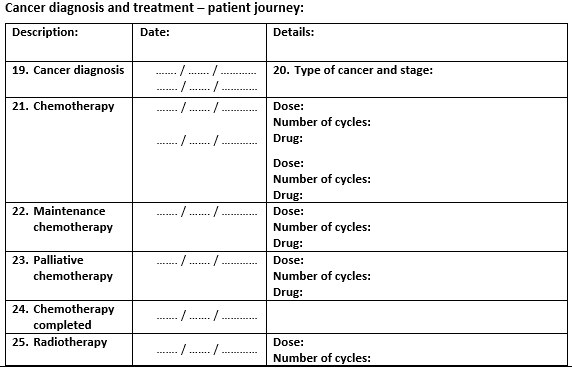
**

**
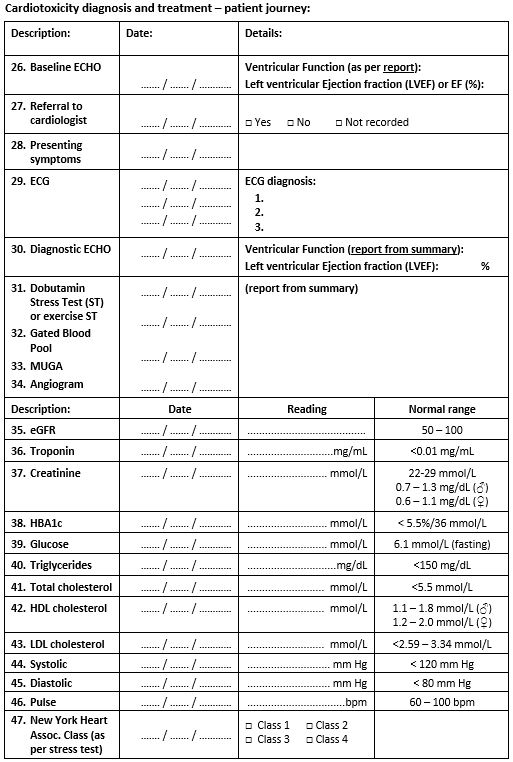
**

**
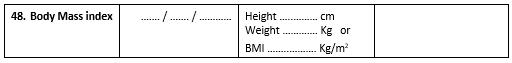
**

**
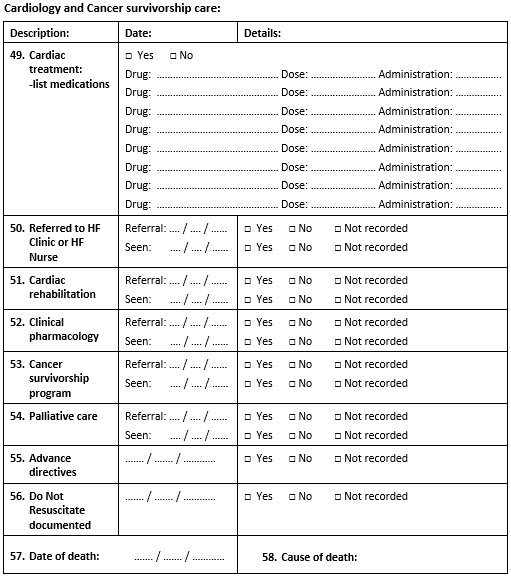
**

**
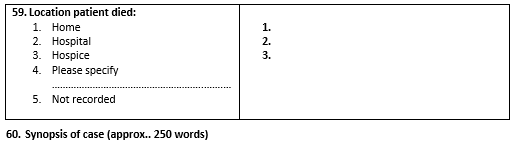
**

Supplement: Supplementary file 1 — Data extraction tool (DET) – MRR. (DOCX 206 kb) [file 40959_2019_46_MOESM1_ESM.docx]
