# Supplementary material for: Cardiotoxicity after cancer treatment: a process map of the patient treatment journey
Source: Cardiooncology. 2019 Aug 22;5:14. doi: 10.1186/s40959-019-0046-5 (PMC7048085; doi:10.1186/s40959-019-0046-5)
Supplement: Supplementary file 2 — Interview schedule. (DOCX 16 kb) [file 40959_2019_46_MOESM2_ESM.docx]

**Additional file 2: Interview schedule**

**Interview schedule**

The aim of the interview is to ascertain the participants’ knowledge, experiences and health service needs. The interview will commence with an assessment of participants’ modifiable and non-modifiable cardiac risk factors.

**Modifiable cardiac risk factors**

1. Can you tell me your cholesterol level?
2. Can you tell me your blood pressure?
3. Waist circumference and body mass index; *or* height and weight?
4. Eating patterns and type of food you like?
5. How many times a week do you eat oily fish?
6. How many times a day do you eat fruit?
7. How many vegetables per day?
8. The sort of physical activity you undertake each day?
9. Minutes of exercise per day?
10. Smoking per day?
11. Ever regularly smoked?
12. Alcohol intake (day/week)?
13. Do you experience anxiety or depression?

**Non-modifiable**

1. Age
2. Gender
3. Indigenous background
4. Family history of cardiovascular disease
5. Diabetes
6. Family history of diabetes
7. Level of social support
8. Family history of high cholesterol

**Cancer history**

1. What sort of cancer were you treated for?
2. When was this diagnosed?
3. Can you remember any of the drugs you were treated with?
4. Were you aware of the risk of cardiac side effects after cancer treatment? If so, how were you made aware of this?

**Cardiac history**

1. How would you describe your current state of health?
2. How would you describe your quality of life?
3. Does your health allow you to do all of the things you would like to do?
4. How does this compare to the way you were before your cancer was diagnosed?
5. What do you understand about your heart condition?

**Health service experiences**

1. Have you had an ECG lately? If so, can you recall the result?
2. Have you had an echocardiogram lately? If so, can you recall the result?
3. Are you taking any medicines for your heart?
4. Can you name them?
5. Have you been experiencing any side effects from your heart medications?
6. What advice has the cardiologist given you about taking a care of your heart?
7. Have you spoken to other health professionals e.g. nurse physio nutritionist or exercise physiologist?
8. What advice have they given you about the care of your heart?
9. What is the cardiac symptom that bothers you most at the moment?
10. Have there been other symptoms that have bothered you previously?
11. What do you now to manage your cardiac symptoms?
12. How did you learn how to manage cardiac symptoms?
13. What health care services do you access at present to look after your heart?

**Health service needs**

1. What health care services do you think you need?
2. Is there any duplication in services?
3. Are there any gaps in services?
4. Do you think you have been supported well by the hospital through your experience with cancer and your heart condition?
5. What advice would you give other patients if they experienced the same heart side effects from their chemotherapy as you have?
6. Is there anything else you can think of that would be important for us to know for our research to help other patients?
